# Machine Learning Predictor of Immune Checkpoint Blockade Response in Gastric Cancer

**DOI:** 10.3390/cancers14133191

**Published:** 2022-06-29

**Authors:** Ji-Yong Sung, Jae-Ho Cheong

**Affiliations:** 1 Department of Laboratory Medicine, Yonsei University College of Medicine, Seoul 03722, Korea; 2 Department of Surgery, Yonsei University College of Medicine, Seoul 03722, Korea; 3 Yonsei Biomedical Research Institute, Yonsei University College of Medicine, Seoul 03722, Korea; 4 Department of Biochemistry & Molecular Biology, Yonsei University College of Medicine, Seoul 03722, Korea

**Keywords:** immune checkpoint blockade, gastric cancer, machine learning, *VCAN*, stem-like type, precision medicine

## Abstract

**Simple Summary:**

This study deals with the identification of signature genes through a model using four machine learning algorithms for two cohorts of bulk and single cell RNA seq to predict immune checkpoint blockade (ICB) response in gastric cancer. Through LASSO feature selection, we identified VCAN as a marker gene signature that distinguishes responders from non-responders.

**Abstract:**

Predicting responses to immune checkpoint blockade (ICB) lacks official standards despite the discovery of several markers. Expensive drugs and different reactivities for each patient are the main disadvantages of immunotherapy. Gastric cancer is refractory and stem-like in nature and does not respond to immunotherapy. In this study, we aimed to identify a characteristic gene that predicts ICB response in gastric cancer and discover a drug target for non-responders. We built and evaluated a model using four machine learning algorithms for two cohorts of bulk and single-cell RNA seq to predict ICB response in gastric cancer patients. Through the LASSO feature selection, we discovered a marker gene signature that distinguishes responders from non-responders. *VCAN*, a candidate characteristic gene selected by all four machine learning algorithms, had a significantly high prevalence in non-responders (*p* = 0.0019) and showed a poor prognosis (*p* = 0.0014) at high expression values. This is the first study to discover a signature gene for predicting ICB response in gastric cancer by molecular subtype and provides broad insights into the treatment of stem-like immuno-oncology through precision medicine.

## 1. Background

Immunotherapy is a revolutionary method that uses the immune system to suppress tumors [1], and it has improved the quality of life of patients with cancer by reducing the side effects of anti-cancer treatment. A more comprehensive perspective of a patient with cancer causes fundamental changes in the assessment of therapeutic efficacy and toxicity [2]. However, the high cost of drugs and their ineffectiveness in some patients are major drawbacks. To overcome these limitations, many studies were conducted to find markers predicting immune checkpoint blockade (ICB) response [3]. Although T lymphocytes have long been recognized for their function in cancer immunosurveillance, the revelation that cancer cells may eventually evade a response from tumor-reactive T cells has sparked efforts to improve the effectiveness of anti-tumor immune response [4]. The most effective molecules of T cell immunological checkpoints are cytotoxic T lymphocyte antigen 4 and programmed cell death 1 [2]. Tumor-associated macrophages and fibroblasts are critical for immunosuppression in relation to the inflammatory tumor microenvironment [5]. Margetuximab, ZW25, and combination strategies involving chemotherapy, human epidermal growth factor receptor 2-targeted treatments, and programmed cell death protein 1 are among the new medications being investigated for gastric cancer treatment [6].

Recently, using machine learning, multi-omics data were used to predict drug responses [7], and gastric cancer data were used to predict ICB responses using a patient stratification algorithm [8]. We previously predicted intra-tumoral heterogeneity with machine learning algorithms using multi-omics data [9]. Machine learning algorithms provide a unique approach for integrating and analyzing omics data, which enables the development of new biomarkers [10]. Owing to its predictive performance and capacity to capture nonlinear and hierarchical characteristics, deep learning algorithms have recently emerged as one of the most promising approaches in multi-omics data analysis [11].

The disadvantage of machine learning studies is that they are limited to bulk omics data; therefore, identifying the cell type as the key to predicting ICB response at the single-cell level is not possible. In this study, we attempted to predict the ICB response using a basic machine learning algorithm and used the approach of a global big data analysis to determine which cell type and ICB response predictions are related at the single-cell level. Our results suggest an effective treatment method by predicting the immune-cancer drug response of patients with gastric cancer through precision medicine.

## 2. Methods

### 2.1. Machine Learning Algorithms

We used four machine learning algorithms to build a model for predicting ICB response. We used R version 4.0.5 with the R package. The labels of tumors of the responder and non-responder groups were assigned 1 and 0, respectively, to classify the tumors according to their ICB response in stomach adenocarcinoma (STAD). For the random forest model [12], glmnet [13], randomForest, ROCR, caret, and e1071 were used. In order to improve the accuracy of classification of the decision trees, the random forest algorithm was used. This is a structure in which multiple trees are created, and a conclusion is reached by combining the predictions of each tree. For the naïve Bayes model [14], glmnet, ROCR, caret, and klaR were used. A naïve Bayes classifier is an algorithm that classifies properties using Bayes’ theorem. Naïve Bayes classifiers are based on the assumption of substantial or naïve independence amongst data point properties. For the neural network model, glmnet, ROCR, and caret were used; for the support vector machine model [15], glmnet, ROCR, caret, e1071, and kernlab were used. A neural network is a series of algorithms that evaluates the relationships between data points, mimicking a network or circuit of biological neurons, or an artificial neural network made up of artificial neurons [16]. A suitable model was found using parameter tuning, and the lambda coefficient was calculated using LASSO [17] feature selection. A 10-fold cross validation was used. To provide a more stabilized result (or one that is more representative of the real-world result), more than 100 iterations were performed, and the average value was used for the AUC value. A 10-fold cross validation was used because (1) the accuracy of data sets can be improved with a small total amount of data; (2) there are more training data sets when classifying only training and testing data than when classifying data into three groups of training/validation/testing; and (3) if the amount of data is small and validation and testing are omitted, a model with poor performance, such as underfitting, is trained.

### 2.2. Single-Cell Analysis

We used Seurat version 4.0 for meta-analysis of single-cell data and classified cell types using marker genes from previous studies [18]. The number of cells (*n* = 5927) used in previously published protocols was maintained, and imputation was performed using Markov Affinity-based Graph Imputation of Cells (MAGIC) [19].

### 2.3. Bulk Sample Meta-Analysis

We predicted ICB response using bulk expression data sets and Tumor Immune Dysfunction and Exclusion (TIDE). METASCAPE [20] was used for gene ontology analysis. For differential gene expression, the “limma” R package was used, and a significant gene with a false discovery rate < 0.001 was selected. For the survival probability in the bulk sample, the web-based tool gepia2 [21] was used, and the drug of the target gene was predicted using the Genomics of Drug Sensitivity in Cancer (GDSC) database [22]. We used the String app [23] in cytoscape [24] for protein–protein interactions (PPIs).

## 3. Results

### 3.1. Prediction of ICB Response of Machine Learning Algorithms

**Figure 1:**
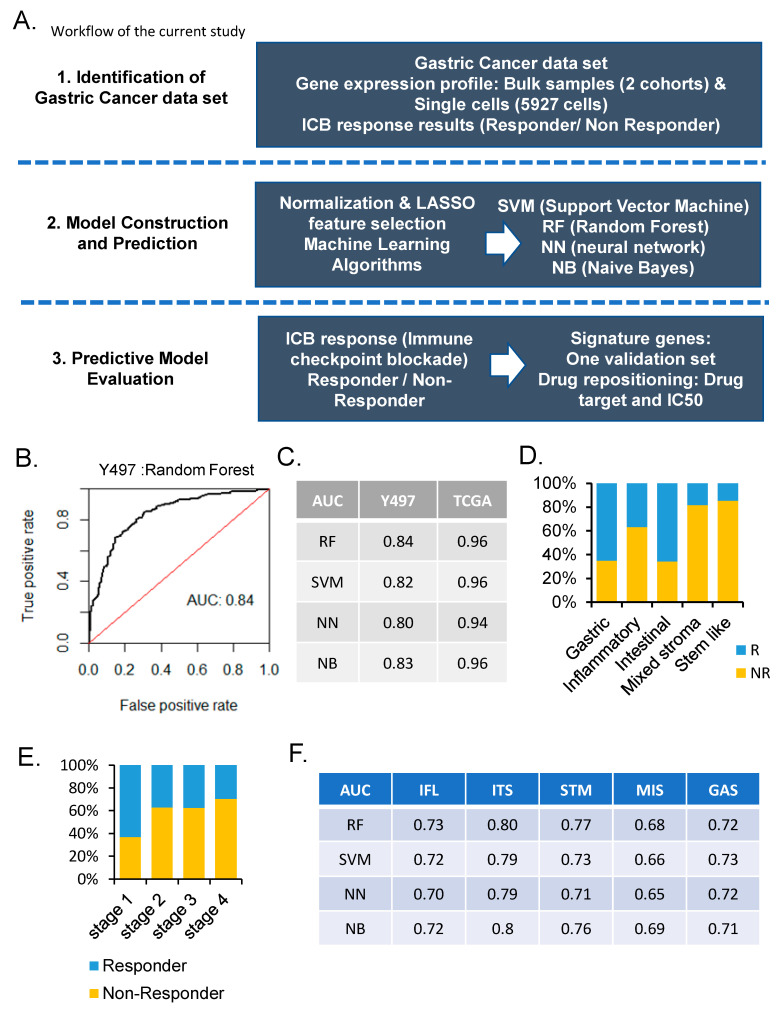
**Machine learning predicts immune checkpoint blockade (ICB) response.** (**A**) Overview of the study workflow and analysis pipeline. (**B**) Receiver operating characteristic curve of predictive performance on random forest in the Yonsei hospital cohort (Y497). (**C**) Performance comparison of classifiers in Y497 and the Cancer Genome Atlas (TCGA) stomach adenocarcinoma (STAD) dataset. (**D**) Bar graph of molecular subtype for ICB response in Y497 (Blue, R, responder; Yellow, NR, non-responder). (**E**) Bar graph of tumor stage of ICB response (Blue, R, responder; Yellow, NR, non-responder). (**F**) Performance comparison of classifiers in molecular subtype of Y497. Molecular subtype: IFL: Inflammatory, ITS: Intestinal, STM: Stem-like, MIS: Mixed stroma, GAS: Gastric.

Predicting the ICB response in gastric cancer is critical. We used bulk expression and single-cell data from two cohorts of gastric cancer and one validation set and the TIDE algorithm [25] for ICB response results (Figure 1A). To build the model, we used four machine learning algorithms (support vector machine, random forest, neural network, and naïve Bayes) and selected the most important genes through LASSO feature selection [9]. The machine learning algorithm was adapted from our previous study [9]. We found a gene signature of 85 genes that can predict the ICB response of patients with gastric cancer, performed validation using 45 Samsung Medical Center (SMC) expression data [26], and evaluated the molecular subtype of our cohort Y497 [27]. Our model showed the highest performance in random forest among the four machine learning algorithms (Figure 1B,C). The Cancer Genome Atlas (TCGA) STAD data set showed excellent performance in comparison with Y497. The TCGA data set had a greater number of genes at 18816 than Y497 had, which was consistent with this trend (Figure 1C). As the number of feature genes increased, an increase in the performance was noticed. Each molecular subtype of gastric cancer had a varied ratio of non-responders to responders (Figure 1D). The stem-like type had the lowest responder ratio, whereas the gastric and intestinal types had similar percentages of responders and non-responders. We investigated the responders and non-responders by the American Joint Committee on Cancer (AJCC) stage and found that stage 4 had the highest number of non-responders (Figure 1E). Depending on the molecular subtype and tumor development and progression, these factors may alter the ICB response. Finally, we examined five different molecular subtypes of gastric cancer to analyze the performance of our machine learning model (Figure 1F). Overall, the performance of each molecular subtype seemed to decrease compared with that of the entire sample; however, the intestinal type showed similar results. In the analysis by molecular subtype, the random forest model showed the best performance. In summary, our machine learning algorithm showed an enhanced performance as the number of gene features and samples increased, particularly in the intestinal molecular subtype.

### 3.2. Prediction of ICB Response According to Single-Cell Types

**Figure 2:**
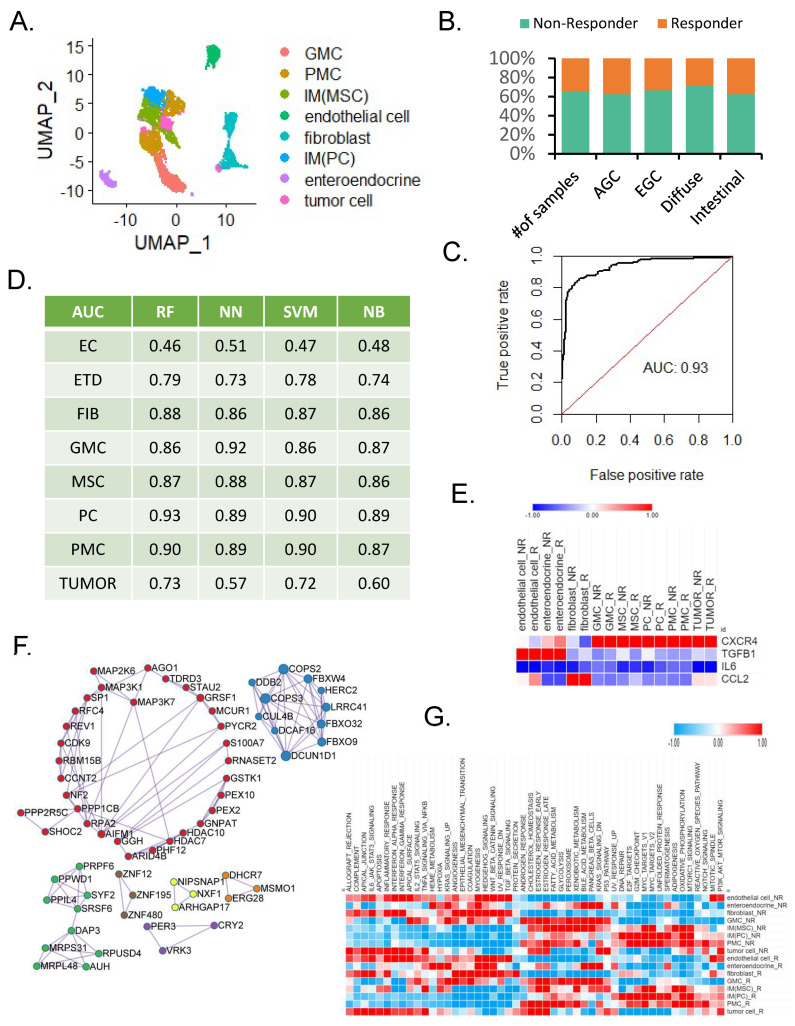
**Comparison of single**-**cell type**-**specific ICB response.** (**A**) Uniform manifold approximation and projection of single-cell types. (**B**) Bar graph of single-cell data of non-responders and responders. (**C**) Receiver operating characteristic curve of predictive performance on random forest in proliferation cells. (**D**) Performance comparison of classifiers in single-cell type. (**E**) Heat map of ICB target genes for ICB response. (**F**) PPI network of LASSO feature selection genes of single cells. (**G**) Heat map of cancer hallmarks of ICB response.

We applied the ICB prediction model built in bulk to compare the performance of single-cell data by cell type. A sample of 23 patients with early and advanced intestinal and diffuse types of gastric cancers was studied [18]. We classified this sample into eight cell types using marker genes from previously published studies (Figure 2A). Non-responders and responders were predicted using TIDE [25] and bulk simulation of single-cell data. More than 60% of the single-cell samples and 70% of the diffuse types were non-responders (Figure 2B). We compared the performance of eight cell types using the four machine learning algorithms and evaluated whether cells other than immune cells were effective for predicting the ICB response in relation to cell types. It showed the highest performance in the random forest of proliferating cells (Figure 2C), and a similar performance pattern was observed in pit mucous cells (Figure 2D). We confirmed the lowest performance in endothelial cells, but the association with ICB response prediction was weak, and the performance was not high in tumor cells. These results are able to extract important cell-type signatures in ICB response prediction. We selected signature genes predicting ICB response through LASSO feature selection in several cells, including fibroblasts (*HBB*, *SHOC2*, *HBA2*), gland mucous cells (*C2orf88*, *IGLL5*, *ANGPTL4*), metaplastic stem-like cells (*SMIM7*, *TMEM80*, *GGPS1*), proliferative cells (*ARL14*, *PIM3*, *IFRD1*), and pit mucous cells (*E4F1*, *TDRD3*, *MTRNR2L1*) (Table S1). In addition, we directly compared the immune checkpoint target genes according to the ICB response of each cell type and confirmed that the difference according to the cell type was larger than the difference between the responder and non-responder. *CXCR4* was highly expressed in most cell types (Figure 2E). We selected 20 signature genes that can predict ICB responses from eight cell types and performed PPI analysis through MCODE, except for the overlapping genes, and vesicle-mediated transport and response to hydrogen peroxide and reactive oxygen species (ROS) were analyzed (Figure 2F). The cancer hallmarks of eight cell types according to the ICB response were different depending on the cell type (Figure 2G).

These results confirmed the common mechanism of ICB response in various cell types.

### 3.3. Molecular Characteristics of ICB Response According to Subtypes of Gastric Cancer

**Figure 3:**
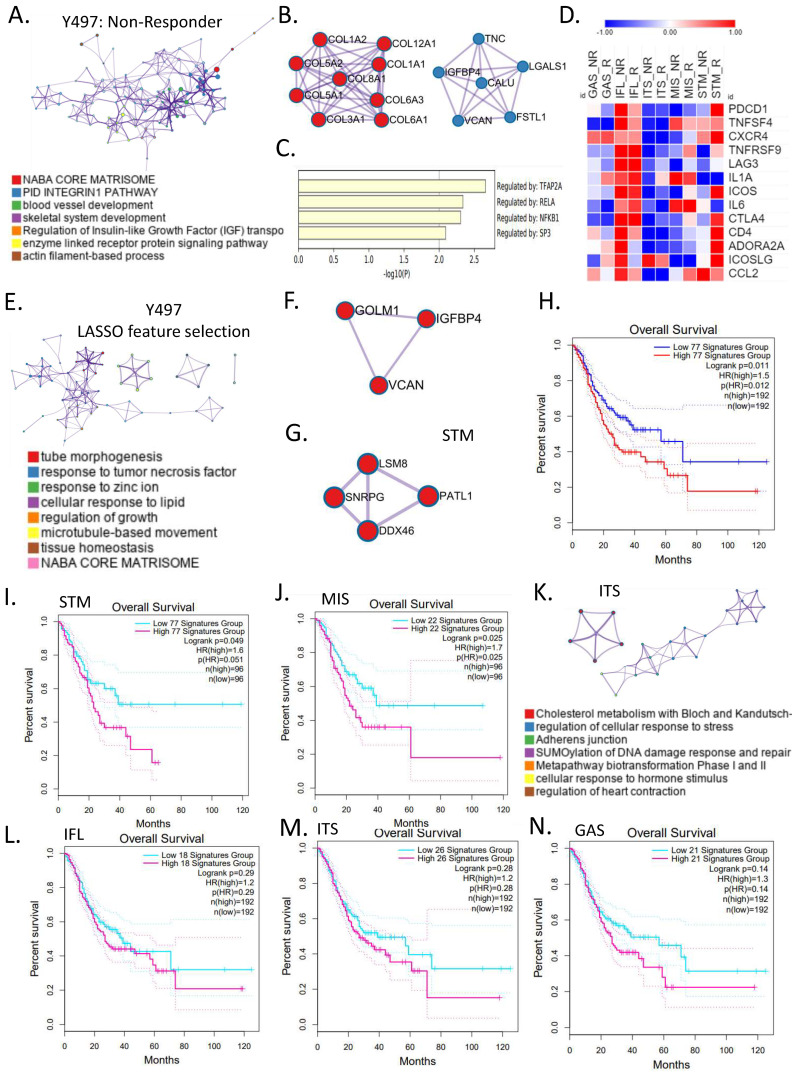
**Meta-analysis of ICB signature genes via LASSO feature selection genes.** (**A**) Network of enriched gene ontology in non-responders of Y497. (**B**) Protein–protein interactions (PPI) in enriched genes in non-responders of Y497. (**C**) Bar graph of transcriptome factors in non-responders of Y497. (**D**) Heat map of ICB target genes for molecular subtype of ICB response in Y497. (**E**) Network of enriched gene ontology in LASSO feature selection genes in Y497. (**F**) PPI of LASSO feature selected genes. (**G**) PPI of LASSO feature selected genes in stem-like type. (**H**) Kaplan–Meier plots of overall survival rates for the high- and low-77 signature genes in TCGA STAD (feature selected genes in Y497). (**I**) Kaplan–Meier plots of overall survival rates for the high- and low-77 signature genes in TCGA STAD (feature selected gene in stem-like type). (**J**) Kaplan–Meier plots of the overall survival rates for the high- and low-22 signature genes in TCGA STAD (feature selected gene in mixed stroma type). (**K**) Network of LASSO feature selected gene ontology in intestinal type of nonresponders of Y497. (**L**) Kaplan–Meier plots of overall survival rates for the high- and low-18 signature genes in TCGA STAD (feature selected gene in inflammatory type). (**M**) Kaplan–Meier plots of overall survival rates for the high- and low-26 signature genes in TCGA STAD (feature selected genes in intestinal type). (**N**) Kaplan–Meier plots of overall survival rates for the high- and low-21 signature genes in TCGA STAD (feature selected genes in gastric type).

In this study, we extracted a gene signature from the ICB response group and explored its drug resistance mechanism. First, through a gene ontology analysis in the responder and non-responder groups of Y497, the naba core matrix, integrin1 pathway, and blood vessel development were enriched in the non-responder group (Figure 3A). Most of these genes were enriched in collagen chain trimerization, naba collagens, syndecan 1 pathway, the regulation of insulin-like growth factor (IGF), and naba core matrix [28] through MCODE PPI [20] (Figure 3B). These genes were found to be regulated by TFAP2A, RELA, NFKB1, and SP3 (Figure 3C). We aimed to determine the different mechanisms involved in ICB response for each molecular subtype. Immune checkpoint target genes were classified into responders and non-responders of molecular subtypes of gastric cancer. The inflammatory type showed a higher ICB target gene expression than other molecular types, and the stem-like type responder group showed a higher expression than the non-responder group (Figure 3D). The intestinal type showed a low ICB target gene expression. We found enrichment in tube morphogenesis, the response to tumor necrosis factor, response to zinc ion, and naba core matrix through the gene ontology analysis of signature genes extracted through machine learning algorithms and LASSO feature selection (Figure 3E) (Table S2). The association between the matrisome with metabolism and immune responses in the refractory type of gastric cancer was determined in our previous study [28]. In the group with a high matrisome, poor prognosis and increased drug resistance were associated with metabolic reprogramming. We analyzed the PPI of the signature that can predict the ICB response through MCODE, which was related to three core genes, *GOLM1*, *IGFBP4*, and *VCAN* (Figure 3F). We analyzed the PPI of signature genes in the ICB response group of stem-like molecular subtypes in the same way, and the four key genes were *LSM8*, *SNRPG*, *PATL1*, and *DDX46* (Figure 3G) (Table S3). In the TCGA dataset, the ICB signature in Y497 demonstrated a poor prognosis at high expression (*p* = 0.011) (Figure 3H). After finding the ICB signatures in five molecular subtypes, we confirmed the prognosis in the TCGA data set. The signatures found in the stem-like and mixed stroma type groups significantly classified patient prognosis in the TCGA data (Figure 3I,J). For signatures of the remaining three molecular subtypes, patient prognosis was not significantly divided in the TCGA dataset (Figure 3L–N). Interestingly, in the intestinal type, genes from the non-responder group were enriched for cholesterol metabolism, regulation of cellular response to stress, and adherens junctions (Figure 3K). In summary, our results indicate signatures for predicting ICB response and each molecular subtype acting towards suppression of the immune response.

### 3.4. Evaluation and Validation of ICB Response Signature Genes

**Figure 4:**
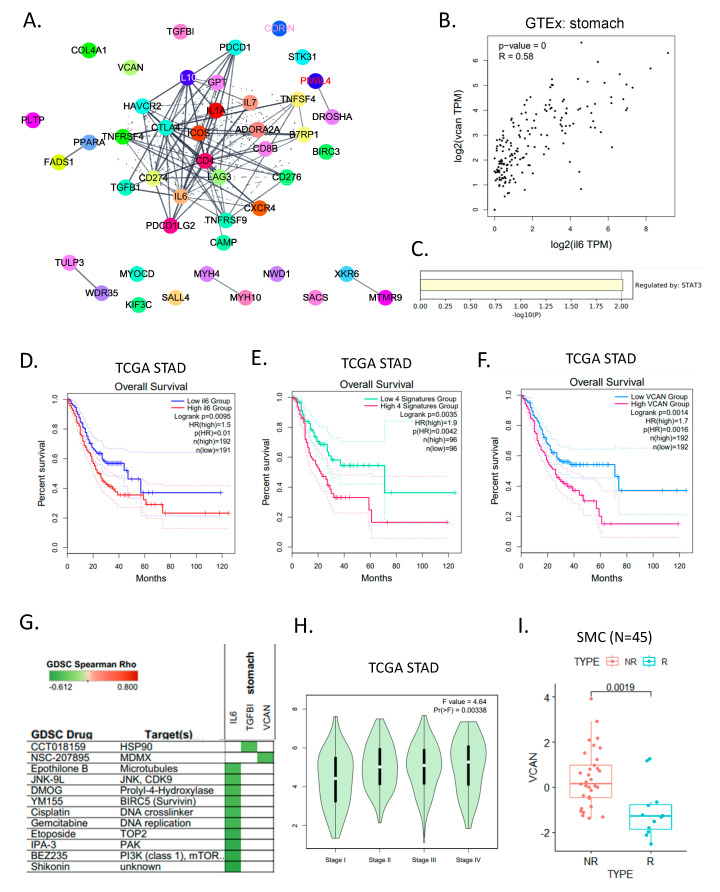
**Validation of feature-selected genes.** (**A**) PPI network of feature-selected and clinically targeted ICB genes. (**B**) Scatter plot of correlation between IL6 and VCAN in the GTEx stomach data set. (**C**) Bar graph of transcriptional factor in feature selected and ICB target genes. (**D**) Kaplan–Meier plots of overall survival rates for the high- and low IL6 expression in TCGA STAD. (**E**) Kaplan–Meier plots of overall survival rates for the high and low top four ranked genes in TCGA STAD. (**F**) Kaplan–Meier plots of overall survival rates for the high and low VCAN expression in TCGA STAD. (**G**) Predicted drugs from Genomics of Drug Sensitivity in Cancer database for IL6, TGFBI, and VCAN. (**H**) Violin plot of VCAN expression in each stage. (**I**) Boxplot of VCAN expression in non-responders (NR) and responders (R) in Samsung Medical Center data (validation set).

We performed LASSO feature selection of responders and non-responders through parameter adjustment using the four machine learning algorithms. Of the original list of 85 genes, 77 genes were identical to the TCGA STAD data set embedded in GEPIA2 (http://gepia2.cancer-pku.cn/#index) (accessed on 29 March 2022), and 8 genes were excluded. Therefore, we analyzed 77 signature genes found through machine learning and the hub genes through the PPI network of 20 clinically targeted ICB genes (Figure 4A). Interleukin 6 (IL6) and IL10 were linked to several candidate target genes, and *VCAN* particularly interacted with the major hub genes. *VCAN* improves cancer cell survival, proliferation, migration, invasion, angiogenesis, treatment resistance, and metastasis in vitro and in vivo [29,30,31].

We confirmed the positive correlation (R = 0.58) between *VCAN* and *IL6* from the stomach GTEx dataset (Figure 4B). The genes identified through the PPI network were regulated by a transcriptome factor called STAT3 (Figure 4C). To determine how these signature genes are clinically related to patient prognosis, we checked the TCGA STAD data set. A high expression of *IL6* (Figure 4D), *VCAN*, and the top four signature genes showed a poor prognosis (Figure 4E,F). We compared drug sensitivity in GDSC for several drug target candidate genes. *IL6* was highly sensitive to shikonin, BEZ235, and IPA-3, and *VCAN* was predicted to be sensitive to NSC-207895 (Figure 4G). We considered *VCAN* over *IL6* as a candidate gene because, although it was not a PPI hub gene, it scored high in all four machine learning algorithms. The parameter selection function in LASSO regression improves prediction accuracy by lowering the regression coefficient. Using the LASSO feature selection method, we acquired a list of genes that were related to the categorization (Table S2). The VCAN gene had the highest coefficient value in the bulk RNA expression dataset when we evaluated the top ranked genes. *VCAN* expression in TCGA STAD data were compared for each stage and was highest in stage 4 (*p* = 0.00338) (Figure 4H). The actual ICB response data from the SMC indicated that *VCAN* was significantly high in the non-responder group (*p* = 0.0019) (Figure 4I). *VCAN* [32] is chondroitin sulfate proteoglycan core protein 2, a mesenchymal stem cell and lineage-specific marker that acts on chondroitin sulfate/dermatan sulfate metabolism, ERK signaling, and glycosaminoglycan metabolism.

These results verify that our machine learning model had good performance and worked well in the validation set.

## 4. Discussion

Studies have been conducted to identify potential therapeutic targets of cancer using omics data in machine learning algorithms. We used a basic machine learning algorithm to perform feature selection and predict the ICB response in gastric cancer. The signature genes [33] have the advantage of predicting the ICB response of patients with gastric cancer and provide general insights for researchers to study the mechanism. We selected a signature gene that discriminates between the ICB responses for each algorithm using bulk and single-cell expression data. In particular, the signature genes from the bulk sample were related to tumor necrosis factor and naba core matrix. The high expression of these genes showed poor prognosis. We selected signature genes for each molecular subtype. In stem-like and mixed stroma types, high expression of the signature gene was associated with poor prognosis, which did not significantly differ between the groups. Our analysis predicted ICB response according to single-cell and bulk types. The performance of tumor cells was not higher than that of other cell types regarding the ICB response, and other cell types were more suitable for the predictive model. Among single-cell types, the random forest model of proliferating cells showed the best performance, and the performance of endothelial cells was poor; however, the relevance to the ICB response prediction model was weak. We confirmed that the signatures for each cell type were significantly related to ROS. Upon analyzing the relationship between predicted signatures and ICB target genes, we confirmed that *VCAN* significantly interacted with them. *VCAN* acts as a mesenchymal stem cell marker, the expression of which was significantly higher in the non-responder group than in the responder group. In summary, our study selected a signature that can predict ICB response with a basic machine learning algorithm using gastric cancer bulk and single-cell expression data. This will enable efficient treatment of patients with precision medicine.

However, there are some limitations to machine learning studies. One of these is deterministic problems. For example, a neural network can find links between inputs and outputs, but it cannot tell you why they are connected. Another issue is the amount of data. Neural networks are complicated designs that necessitate a large quantity of training data to provide useful results. As the design of a neural network grows larger, so too does the amount of data that it requires. Thus, an immunotherapy system’s biology, as well as its description, are beyond the comprehension and scope of neural networks.

## 5. Conclusions

Predicting the immunotherapy response in patients with gastric cancer is an important issue, particularly in stem-like molecular types, which show drug resistance and several non-responders. Our results can be used in clinical practice for predictive responses to ICB in patients with gastric cancer. Moreover, by suggesting drug targets, combination therapy can be performed according to ICB response, which contributes to drug development and precision medicine.

## Data Availability

Not applicable.

## Supplementary Materials

The following supporting information can be downloaded at: https://www.mdpi.com/article/10.3390/cancers14133191/s1, Supplementary Table S1. LASSO feature selection genes in single-cell data; Supplementary Table S2. LASSO feature selection genes in Y497; Supplementary Table S3. LASSO feature selection genes in stem-like type.

## Abbreviations

ICB	immune checkpoint blockade
STAD	stomach adenocarcinoma
PPI	protein–protein interaction
TIDE	Tumor Immune Dysfunction and Exclusion
GDSC	Genomics of Drug Sensitivity in Cancer
TCGA	The Cancer Genome Atlas
IGF	insulin-like growth factor
IL	interleukin
